# Description of Maternal Smoking Status Before and After Pregnancy: A Longitudinal, Community-Based Cohort Study

**DOI:** 10.2188/jea.JE20180187

**Published:** 2020-07-05

**Authors:** Katsuya Ueda, Naomi Kitano, Kohta Suzuki

**Affiliations:** 1 Wakayama Medical University Hospital, Wakayama, Japan; 2 Research Center for Community Medicine and Department of Public Health, School of Medicine, Wakayama Medical University, Wakayama, Japan; 3 Department of Health and Psychosocial Medicine, Aichi Medical University School of Medicine, Aichi, Japan

**Keywords:** maternal smoking, pregnancy, cessation, perinatal, community-based cohort study

## Abstract

**Background:**

Maternal smoking during pregnancy is a major risk for adverse perinatal outcomes, as well as children’s health status. Thus, it is important to describe maternal smoking status during pregnancy and child-rearing to devise better intervention strategies. However, there have been no longitudinal studies to describe the status. Thus, in this study, we aimed to describe maternal smoking status during pregnancy and child-rearing based on population-based maternal and child health information. Moreover, we explored the factors associated with maternal smoking relapse after delivery.

**Methods:**

We performed a survey of 1,220 mothers in a Japanese rural area who responded to a questionnaire upon registration of their pregnancies. When their children received health checkups at 4, 18, and 36 months of age, maternal smoking status was also surveyed. We then performed multiple logistic regression analysis to explore factors associated with maternal smoking relapse after delivery.

**Results:**

Ultimately, the total number of mothers with data available for longitudinal analysis was 727 (59.6%). At the time of pregnancy registration, there were 74 current smokers (10.2%) and 176 former smokers (24.2%). Among them, 59 (33.5%) relapsed after delivery. Under 28 years of maternal age at pregnancy registration (OR 2.6; 95% CI, 1.2–5.4) was associated with maternal smoking relapse after delivery.

**Conclusions:**

Longitudinal analyses showed that about 60% of mothers who smoked before and after delivery failed smoking cessation. In addition, younger mothers were significantly likely to relapse smoking after delivery.

## INTRODUCTION

The concept of ‘developmental origins of health and disease’ has been increasingly applied to the field of perinatal and pediatric epidemiology. Based on this theory, the idea that maternal smoking during the perinatal period plays an important role in childhood growth and development has been proposed^1^ because some perinatal complications, such as premature membrane rupture, placental abruption, preterm delivery, and low birth weight, are caused by maternal smoking during pregnancy.^2^ Actually, it has been suggested that sudden infant death syndrome,^2^^,^^3^ as well as childhood obesity and delayed development,^4^^–^^6^ are also associated with maternal smoking during pregnancy.

On the other hand, maternal smoking after delivery shortens the breastfeeding period,^7^ and continued smoking during breastfeeding may expose infants to passive smoking and to nicotine transferred via breast milk. Nicotine in breastfed infants is associated with apnea, changes in sleep/wake patterns, and infant iodine deficiency because of the reduced iodine supply in breast milk.^8^ Associations between childhood passive smoking and asthma,^9^ middle ear diseases,^10^ and caries have also been previously reported.^11^^,^^12^

The maternal smoking rate during pregnancy in Japan is reported every 10 years by the Ministry of Health, Labour and Welfare. Accordingly, the nationwide maternal smoking rates during pregnancy were 5.6%, 10.0%, and 5.0% in 1990, 2000, and 2010, respectively.^13^ Furthermore, the Japan Environment and Children’s Study also found maternal smoking rates during early pregnancy to be 5% and 9% among total participants and mothers younger than 25 years old, respectively.^14^ Although the maternal smoking rate during pregnancy has decreased from 2000 to 2010, it remains a major public health concern. Additionally, the Longitudinal Survey of Newborns in the 21st Century (2010 cohort) also reported the rates of maternal smoking 6 months after delivery to be 7.0% in total participants, 22.6% in mothers younger than 20 years, and 16.9% in mothers aged 20–24 years.^15^ The authors of this report also suggested that maternal smoking in younger mothers was a particularly major concern.

Many studies of maternal smoking during pregnancy and after delivery have focused on associated risk factors.^16^^–^^18^ However, only a few studies examined individual smoking statuses longitudinally during and after pregnancy.^19^^–^^21^ Kahn et al reported an association between maternal educational background and smoking during and after pregnancy.^19^ Moreover, Hauge et al found that maternal psychological stress and interpersonal relationship problems can be related to both maternal smoking during pregnancy, as well as smoking relapse after delivery.^20^ In Japan, Suzuki et al reported that women who stopped smoking after the onset of pregnancy were more likely to relapse after delivery than women who had stopped smoking before becoming pregnant.^21^

Generally, motivation for the cessation of smoking is associated with higher income, younger age, fewer cigarettes smoked per day, being married, and a higher educational background.^22^^,^^23^ Moreover, Alves et al reported that maternal older age, primipara, fewer cigarettes smoked per day, continuous cohabitation with a partner, pregnancy after cessation of smoking, breastfeeding beyond 52 weeks of age, and childhood asthma or rhinitis might be associated with sustained abstention from smoking among new mothers.^24^ However, few epidemiological studies have examined the factors associated with smoking relapse after pregnancy.

Because city administration offices across Japan are notified of confirmed pregnancies, and based on periodically scheduled infancy and childhood health check-ups under the Maternal and Child Health Act, every municipal government has data available on maternal smoking status during and after pregnancy. Therefore, our study primarily aimed to describe the longitudinal maternal smoking status and to determine the smoking rates during these periods. The secondary aim of this study was to examine factors that are associated with maternal smoking relapse after delivery, using the same dataset.

## METHODS

### Study population and setting

This was a population-based longitudinal survey of 1,220 mothers who registered their pregnancy at city office from October 2004 through March 2010 in Gobo City, Wakayama Prefecture, Japan. Based on the 2015 census data, Gobo city has 43.78 km^2^ area and the number of residents was 24,801, including 3,057 who were younger than 15 years old.

### Study design

We used maternal and child health information from the Gobo City administration office. Mothers or their partners are asked to complete questionnaires at the time of registration of their pregnancy and during health check-ups for their children at 4, 18, and 36 months of age (ie, four questionnaires in total). We extracted smoking data collected from these questionnaires and constructed an anonymized electronic database with the cooperation of the Gobo City administration office.

### Data collection

In all four surveys, information on the maternal age, number of children, and personal and family history of smoking was collected from all the participants. For mothers who were current smokers, we collected information on the amount of smoking, age at which they started smoking, main location of smoking, and willingness to cease the habit. Furthermore, information on smoking time spans was collected from mothers who were former smokers. Additional surveys on smoking locations were conducted for participants who had smoking family members. Responses to questionnaires (completed by the mothers or their partners) were examined at the 4-, 18-, and 36-month health check-ups.

We used six questions to determine the knowledge of expectant mothers regarding the adverse effects of maternal smoking during pregnancy on its outcomes. The questions included the association between maternal smoking and preterm delivery, spontaneous abortion, premature rupture of membranes, placental abruption, low birth weight, congenital anomalies, and sudden infant death syndrome. Although the number of questions was six, there was a question that asked about two adverse effects; therefore, there was a difference of these numbers.

### Data analysis

#### Outcome variables

Maternal smoking status was divided into three categories: never smoker, former smoker, and current smoker. There were some inconsistencies where mothers stated they were “never smokers” on some questionnaires despite responding that they were “former smokers” or “current smokers” on others. If it was possible to derive accurate information based on their collective responses, we revised their answers and included their ‘corrected’ data in our analysis; otherwise, they were excluded from the study. An example of this situation is as follows: Some mothers stated that they had never smoked when they first registered their pregnancies; however, they stated that they were “current smokers” at their infants’ 4-month health check-up and that they had started smoking before becoming pregnant. Therefore, we changed the smoking status at registration of pregnancy from “never smoker” to “current smoker.”

#### Statistical analysis

We report descriptive statistics, including the age of expectant mothers, number of children, smoking status of mothers, smoking status of family members, and degree of knowledge of the adverse effects of maternal smoking during pregnancy on the outcomes of pregnancy. To assess the degree of knowledge, we calculated the proportion of correct responses to the six knowledge questions.

**Figure 1.**
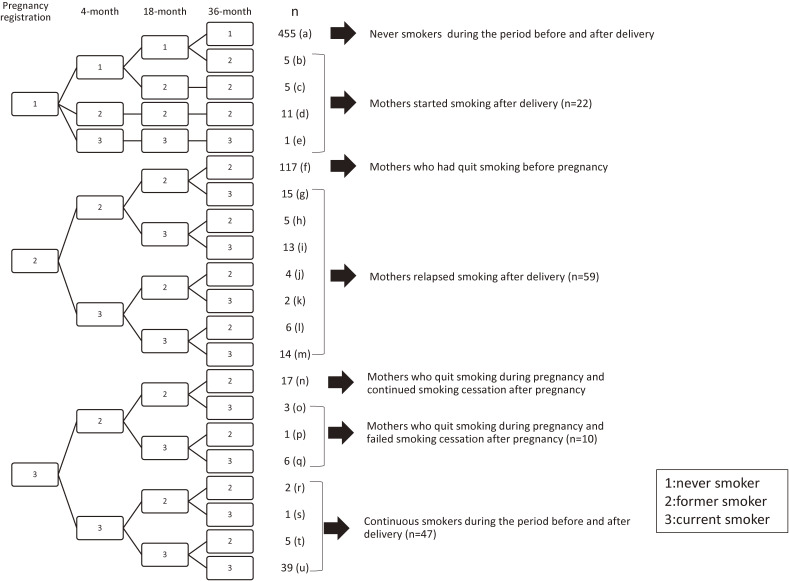
Tree diagram to describe maternal smoking status at pregnancy registration and health check-ups for their children at 4, 18, and 36 months of age (1: answer of “never smoker”, 2: answer of “former smoker”, 3: answer of “current smoker”)

First, we longitudinally described maternal smoking status before and after delivery under the following definitions (Figure 1). The mothers who were “never smoker” at registration of pregnancy were classified into “Never smoker during the period before and after delivery” (mothers who answered “never smoker” at all surveys) and “Mothers started smoking after delivery” (mothers who answered “former smoker” or “current smoker” at least one survey after delivery). The mothers who were “former smoker” at registration of pregnancy were classified into “Mothers who had quit smoking before pregnancy” (mothers who answered “former smoker” at all surveys) and “Mothers relapsed smoking after delivery” (mothers who answered “current smoker” at least one survey after delivery). Regarding to “Mothers relapsed smoking after delivery”, we analyzed the timing that they relapsed smoking. The mothers who were “current smoker” at registration of pregnancy were classified into “Continuous smoker during the period before and after delivery” (mothers who answered “current smoker” at the 4-month health check-up, regardless appearance their answers of “former smoker” at the 18-, and 36-month health check-ups.) and “Mothers who quit smoking during pregnancy” (mothers who answered “former smoker” at the 4-month health check-up). Regarding “Mothers who quit smoking during pregnancy”, we further divided this group into two categories: “Mothers who continue smoking cessation after pregnancy” (mothers who answered “former smoker” at all surveys after delivery) and “Mothers who failed to continue smoking cessation after pregnancy”.

#### Analyses of factors that are associated with maternal smoking relapse after delivery

We conducted multivariable logistic regression analyses to examine the factors associated with maternal smoking relapse after delivery among the participants who quit smoking before pregnancy.

We used the following data, derived at the time of registration of pregnancy, as variables that are potentially predictive of maternal smoking relapse after delivery: maternal age, number of children, smoking status of family members, and knowledge of the effects of smoking during pregnancy on its outcomes. Maternal ages at registration of pregnancy were divided into two categories using the median (28 years) as a cutoff: ≤28 and ≥29 years. The numbers of children at registration of pregnancy were divided into two categories: 0 (primipara) and ≥1 (multipara). The smoking status of family members at registration of pregnancy was categorized as “smoking (yes)” and “non-smoking (no)”. The number of correct answers to questions regarding knowledge of the effects of maternal smoking during pregnancy were divided into two categories using the median value of 3: ≤3 and ≥4.

All analyses were performed using SPSS Statistics version 20.0 for Windows (IBM Corp., Armonk, NY, USA). Two-sided *P*-values <0.05 were considered statistically significant.

## RESULTS

### Study participants

Of the 1,220 mothers who participated in this study, 398 were excluded because of missing data (including changes of residence) and 45 were excluded because their questionnaires were completed by their partners. There were some contradictions about maternal smoking status in 131 (10.7%). Of these, 50 were excluded because of inconsistent answers regarding maternal smoking status, 81 were logically corrected and included the analysis. Therefore, the total number of mothers with data available for longitudinal analysis was 727 (59.6%).

On the other hand, the numbers of women with available data for cross-sectional analyses were 884 (72.5%) at registration of pregnancy, 1,008 (82.6%) at the 4-month health check-up, 1,028 (84.3%) at the 18-month health check-up, and 1,067 (87.5%) at the 36-month health check-up. The number of women who smoked were 74 (10.2%) at registration of pregnancy, 74 (10.2%) at 4-month health check-up, 90 (12.4%) at 18-month health check-up, and 94 (12.9%) at 36-month health check-up.

### Characteristics of mothers at registration of pregnancy

Table 1 shows the characteristics of mothers at the time of pregnancy registration. The median age of mothers at this stage was 29 years (range, 16–42 years). With regard to maternal parity (birth order of indexed newborns), 289 (39.8%) were first births and 300 (41.3%) were second births. The median knowledge score of the effect of smoking during pregnancy on its outcomes was 3 (range, 0–6 points). Furthermore, the number of cases of smoking among family members at registration of pregnancy was 476 (65.5%).

**Table 1.** Characteristics of study participants at pregnancy registration (*n* = 727)

Variables	*n* (%)
Age of expectant mothers, years	
≤19	16 (2.2)
20–24	133 (18.3)
25–29	269 (37.0)
30–34	228 (31.3)
35–39	74 (10.2)
≥40	7 (1.0)
Parity	
0 (nulliparity)	289 (39.8)
1	300 (41.3)
2	113 (15.5)
3	20 (2.8)
4	4 (0.6)
5	1 (0.1)
Knowledge score regarding the effects of smoking during pregnancy on pregnancy outcomes	
0	40 (5.5)
1	40 (5.5)
2	95 (13.1)
3	157 (21.6)
4	141 (19.4)
5	113 (15.5)
6	141 (19.4)
Smoking family members at the time of pregnancy registration	
Yes	476 (65.5)
No	251 (34.5)

Regarding smoking status, the mean number of cigarettes smoked at the registration of pregnancy and at relapse smoking were 12.4/day and 10.3/day, respectively.

### Longitudinal maternal smoking status and smoking rates at each survey (*n* = 727)

Figure 1 shows the longitudinal smoking statuses of mothers. Each number of mothers in Figure 1 was alphabetically indicated by a to u. First, the number of never smokers during the period before and after delivery was 455 (a; 62.6%). Then, the number of mothers who experienced smoking during the same period was 272 (b+c+d+e+f+g+h+i+j+k+l+m+n+o+p+q+r+s+t+u; 37.4%). Among them, the number of mothers who experienced smoking from pregnancy registration to the 36-month health check-up (mothers without “never smokers during the period before and after delivery” and “mothers who had quit smoking during the period before and after pregnancy”) was 155 (b+c+d+e+g+h+i+j+k+l+m+n+o+p+q+r+s+t+u; 21.3%).

There were 176 (f+g+h+i+j+k+l+m; 24.2%) former smokers at pregnancy registration. Among them, there were 59 relapse smokers after delivery (g+h+i+j+k+l+m; 33.5%). Regarding to the timing of smoking relapse among them, 26 (j+k+l+m) were before 4 months after delivery, 18 (h+i) were before 18 months after delivery, and 15 (g) were before 36 months after delivery.

The number of mothers who experienced smoking before and after delivery was 116 (g+h+i+j+k+l+m+o+p+q+r+s+t+u; 16.0%). Among them, 69 (g+h+i+j+k+l+m+o+p+q; 59.5%) failed smoking cessation during this study period.

### Multivariable analyses of factors associated with maternal smoking relapse after delivery (*n* = 176)

Table 2 shows the factors that were found to be associated with maternal smoking relapse after delivery. Age of mother at pregnancy registration (OR 2.6; 95% CI, 1.2–5.4) was associated with maternal smoking relapse after delivery. Other factors were not statistically associated with maternal smoking relapse after delivery.

**Table 2.** Logistic regression analysis to explore factors associated with maternal smoking relapse after delivery (*n* = 176)

	Population at risk, *n* (%)	Mothers relapsed smoking after delivery, *n* (%)	Logistic regression analyses			

			Crude		Adjusted	
	
All	176 (100)	59 (100)	OR (95% CI)	*P*-value	OR (95% CI)	*P*-value
Age of mother at pregnancy registration						
≥29 years	64 (36.4)	14	Reference	0.015	Reference	0.012
≤28 years	112 (63.6)	45	2.4 (1.2–4.8)		2.6 (1.2–5.4)	
Parity						
0	89 (50.6)	28	Reference	0.558	Reference	0.105
≥1	87 (49.4)	31	1.2 (0.64–2.3)		1.8 (0.88–3.7)	
Smoking family members at the time of						
No	36 (20.5)	8	Reference	0.112	Reference	0.195
Yes	140 (79.5)	51	2.0 (0.85–4.7)		1.8 (0.74–4.4)	
Knowledge score regarding the effects						
≥4	99 (56.3)	30	Reference	0.306	Reference	0.251
≤3	77 (43.7)	29	1.4 (0.74–2.6)		1.5 (0.75–3.0)	

CI, confidence interval; OR, odds ratio.

## DISCUSSION

This study investigated the longitudinal maternal smoking status from pregnancy to 3 years post-delivery using information collected by a city administrative office in a Japanese rural area. Among the participants who answered all four surveys, approximately 40% of mothers had smoked.

Among 176 mothers who were former smokers at the time of pregnancy registration, more than 30% of mothers relapsed smoking after delivery. In addition, younger mothers were likely to relapse smoking after delivery. Also, about 60% of mothers who smoked before and after delivery failed smoking cessation.

In this study, the maternal smoking rate at pregnancy registration was 10.2%. Previously reported smoking rates were 5.0% in a nationwide survey and 7.5% in a local survey.^13^^,^^14^^,^^21^ Therefore, these results suggest that the maternal smoking rate in the geographical area where our study was conducted is relatively higher than that in Japan overall. One reason for this difference between studies might be that mothers who live in this rural area are younger than the country’s overall average. The relatively high smoking rate in younger women is an emerging public health hazard in Japan, as well as in other Asian countries.

Next, among mothers who smoked after delivery, more than half of them were former smokers. This result suggests that preventing maternal smoking relapse leads to decrease in overall rate of smoking after delivery. Moreover, because younger mothers were likely to relapse maternal smoking, it might be effective to conduct prevention programs for relapse smoking among younger generations.

### Strengths and limitations of the study

Although it yielded some significant findings, our study nevertheless had some limitations. First, the fact that we used questionnaires to collect information on smoking status leaves open the possibility that some smokers might not have provided accurate responses. However, questionnaires are considered a valid method for determining the smoking status of pregnant women^25^; therefore, the effect of this limitation on our results might not be large. Additionally, because this study was performed only in a Japanese rural area, the results may not necessarily be generalizable. Moreover, we were unable to examine other factors that were previously found to be smoking-related, such as socioeconomic status (educational history and annual income), marital and cohabitation status, and physical and psychological stress.^19^^,^^20^ Finally, the reliability of using six questions to evaluate the knowledge of the effect of maternal smoking during pregnancy on the fetus was not validated.

Nevertheless, this study has several strengths. First, because information on smoking status was collected by administrative offices, such as maternal and child health centers, our results might be accurately reflected the general maternal smoking status in this city. Although some previous studies longitudinally examined maternal smoking status during the pregnancy and child-rearing periods, we collected more detailed information regarding maternal smoking status. Also, there was no previous survey of maternal smoking status that continued to follow up until the 36-month health check-up in Japan. These results might contribute to prevention of children’s exposure to second-hand smoke. Public health nurses employed at administrative offices could develop a pamphlet that describes the main implications of our results, as well as those from studies conducted in other geographic locations, to encourage mothers to stop smoking.

### Conclusions

Maternal smoking rates in the Japanese rural area covered in our study were 10.2% and 37.4% at the time of registration of pregnancy and during the perinatal period, respectively. Longitudinal analyses showed that about 60% of mothers who smoked before and after delivery failed smoking cessation. In addition, younger mothers were significantly more likely to relapse smoking after delivery than older mothers. Our data could assist community healthcare providers in encouraging mothers to stop smoking or to maintain smoking cessation.
